# Identification of novel maize miRNAs by measuring the precision of precursor processing

**DOI:** 10.1186/1471-2229-11-141

**Published:** 2011-10-20

**Authors:** Yinping Jiao, Weibin Song, Mei Zhang, Jinsheng Lai

**Affiliations:** 1 State Key Laboratory of Agrobiotechnology; National Maize Improvement Center; Department of Plant Genetics and Breeding, China Agricultural University, Beijing, 100193, China

## Abstract

**Background:**

miRNAs are known to play important regulatory roles throughout plant development. Until recently, nearly all the miRNAs in maize were identified by comparative analysis to miRNAs sequences of other plant species, such as rice and *Arabidopsis*.

**Results:**

To find new miRNA in this important crop, small RNAs from mixed tissues were sequenced, resulting in over 15 million unique sequences. Our sequencing effort validated 23 of the 28 known maize miRNA families, including 49 unique miRNAs. Using a newly established criterion, based on the precision of miRNA processing from precursors, we identified 66 novel miRNAs in maize. These miRNAs can be grouped into 58 families, 54 of which have not been identified in any other species. Five new miRNAs were validated by northern blot. Moreover, we found targets for 23 of the 66 new miRNAs. The targets of two of these newly identified miRNAs were confirmed by 5'RACE.

**Conclusion:**

We have implemented a novel method of identifying miRNA by measuring the precision of miRNA processing from precursors. Using this method, 66 novel miRNAs and 50 potential miRNAs have been identified in maize.

## Background

MiRNAs are known to play crucial roles in the regulation of gene expression in plants [1], including functions such as, leaf polarity, auxin response, floral identity, flowering time, and stress response [2-7]. MiRNAs are typically ~21 nucleotides in length. In plants, miRNA genes are transcribed by RNA polymerasell into primary miRNA transcripts (pri-miRNA) which can form imperfect stem-loop secondary structure [8,9]. Then the pri-miRNAs are trimmed and spliced into miRNA/miRNA* duplex by Dicer-like1 (DCL1) with the help of dsRNA binding protein HYL1 and dsRNA methylase HEN1 [1,10-12]. The length of the pre-miRNAs in plants ranges from about 80-nt to 300-nt, and is more variable than in animals. After being transported to the cytoplasm, the mature miRNAs can match to the corresponding target mRNAs through RNA-induced silencing complex (RISC) and the miRNA* are thought to be degraded [1,13]. MiRNAs regulate their target mRNA either by cleaving in the middle of their binding sites or by translational repression [14,15]. The plant miRNAs are highly complementary to their targets with about 0~4 nucleotides mismatches [1].

The majority of miRNAs were originally discovered through traditional Sanger sequencing of small RNA pools [16-18]. With the advent of second (next) generation sequencing technology, the rate of miRNA discovery increased dramatically [19-21]. However, due to the complexity of small RNA population, identification of miRNAs from the small RNA pools of sequencing product was not trivial. Typically, genomic sequences matched to all the small RNA with a length of 19~22-nt were extended upstream and downstream to get a collection of candidate precursors. Their secondary structures were then checked using a number of criteria with Minimum Free Energy (MFE) as the most important one [17,19-21]. The presence of miRNA* has been regarded as a golden standard to reliably annotate a novel miRNA. Nevertheless, miRNA* have only been reported to be showed up with mature miRNA around 10% of the time [22]. As miRNAs can be enriched in certain genomic regions, a clustering algorithm was sometimes used for miRNA identification from large scale small RNA sequencing data. In these studies [23-25], hotspots of small RNA generation were identified if they match with multiple known miRNAs; individual hairpin sequences within these hotspots were subsequently checked to see whether some of them could be qualified as miRNAs.

As many miRNAs are conserved among different organisms, sequences of miRNAs found in one species can be used to identify corresponding miRNAs in other species through comparative analysis [6,26]. However, not all the miRNAs are conserved across different organisms. Direct prediction of potential miRNAs, based on the characteristics of miRNA precursors, has been shown to be a useful approach to identify miRNAs for any organisms, provided that there are a large amount of genomic sequences available [27]. However, as millions, even billions of inverted repeat sequences exist in complex genomes, candidate miRNAs identified just based on computational prediction often show a high rate of false positive.

Maize is an important crop as well as a model of plant genetics. A number of miRNAs with specific function have been reported in maize. The miR172 was reported to target *APETALA2 *floral homeotic transcription factor that is required for spikelet meristem determination [28]. Also, miR172 functions in promoting vegetative phase transition by regulating the APETALA2-like gene *glossy15 *[29]. The expression of *teosinte glume architecture1 *(*tga1*), which plays an important role in maize domestication, is regulated by miR156 [28]. The miR166 has been found to target a class III homeodomain leucine zipper (HD-ZIPIII) protein that acts on the asymmetry development of leaves in maize [30].

There are a total of 84 unique maize mature miRNAs belonging to 28 miRNA families in the current version of miRBase (release 17) [31]. These 84 miRNAs are the products of 167 precursors. All of these miRNAs were originally identified by searching with known miRNA from other plant species, such as *Arabidopsis *and rice [31-35]. Recently, 150 mature miRNAs from 26 families were validated by Illumina sequencing [34]. To do *de novo *identification of new miRNAs in maize, we have sequenced small RNAs from mixed tissues, tissues of endosperm and embryo using a next generation sequencing system. Moreover, a new method of identifying novel miRNAs, by measuring the precision of miRNA processing from their precursors, was employed. This method, conceptually proposed by Meyer *et al*., holds that the precise processing from precursor is both necessary and sufficient criterion for miRNA annotation [36]. We report here the establishment of such a method of identifying miRNAs by measuring the precision of miRNA processing from precursors. This method has resulted in 66 newly identified miRNAs and 50 potential miRNAs in maize. Of the 66 newly identified miRNAs, 62 belong to 54 families that have not been identified before in any other organisms.

## Results

### Sequencing of maize small RNAs

**Figure 1:**
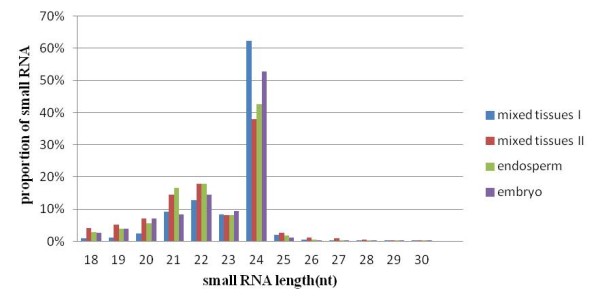
**Small RNA length distribution from four separate sequencing runs**.

In order to identify novel miRNAs from maize, four different small RNA samples (two from mixed tissues, one from embryo and another from endosperm) of B73 inbred line were sequenced. The sequencing effort resulted in over 43 million signatures with a length of 18~30nt, representing over 15 million unique sequences (Table 1). The overall size distribution of the sequenced reads from all four sequencing effort were very similar, with the 24-nt class being the most abundant, followed by 22-nt and 21-nt classes (Figure 1). Such a size distribution is consistent with recent report that 22-nt siRNAs were specifically enriched in maize compared with other plants [37,38]. Although over 43 million sequences were generated, a large number of signatures were only sequenced once, suggesting that maize has a very complex small RNA composition. The percentages of small RNAs sequenced once in four samples were 81.8% (2, 997, 412) and 77.9% (3, 227, 436) in two mixed tissues, 77.5% (5, 339, 164) in endosperm and 78.6% (3, 003, 817) in embryo, respectively. As in other small RNA sequencing efforts, there was a small portion of distinct signatures that matched to mitochondria or chloroplast genomes. In the four independently sequenced samples, there were 4.7%, 5.9%, 7.2% and 19% total signatures that respectively represent 0.26%, 0.50%, 0.49% and 1.2% unique reads matched to non-coding RNAs including tRNA, rRNA, snRNA, snoRNA (Table 2).

**Table 1:** Summary of small RNA sequencing

	No. of reads generated	No. of unique reads	No. of unique reads matched to genome
mixed tissues I	6, 823, 490	3, 664, 019	3, 445, 495
mixed tissues II	11, 978, 592	4, 143, 803	4, 133, 620
embryo	14, 812, 427	6, 886, 540	6, 879, 213
endosperm	9, 567, 504	3, 823, 033	3, 298, 557
total	43, 182, 013	15, 387, 312	15, 220, 296

**Table 2:** Summary of signatures matched to various RNAs

	Mixed tissues I		Mixed tissues II		Embryo		Endosperm	
	Unique reads	Total reads	Unique reads	Total reads	Unique reads	Total reads	Unique reads	Total reads
non_coding RNA	9, 739	322, 288	20, 836	714, 095	34, 177	1, 066, 978	46, 362	1, 825, 974
chloroplast	7, 584	31, 673	50, 750	1, 006, 833	24, 077	43, 271	4, 627	9, 533
mitochondirial	8, 986	29, 197	21, 579	134348	20, 660	31, 359	9, 926	13, 845

### Validation of known maize miRNAs in miRBase

There are a total of 84 unique mature miRNA sequences belonging to 28 miRNA family in the current miRBase for maize. All these miRNAs were identified by computational method based on sequence conservation using sequences of known miRNAs of other species [31-34]. Out of the 84 unique miRNA sequences, 49 can be confirmed by our sequencing effort, while 25 were detected in all four libraries. Except for zma-miR393, zma-miR1432, zma-miR408, zma-miR482 and zma-miR395, 23 of 28 known maize miRNA families had members detected in at least one of the four sequenced libraries. Some of the conserved miRNAs showed very high abundances in our sequenced libraries, for example, zma-miR156a, b, c, d, e, f, g, h and i had more than 20, 000 reads in our four samples (Table 3).

**Table 3:** Expressional abundance of the known miRNAs calculated in Reads per Million

Family	miRNA name	mixed tissues I	mixed tissues II	embryo	endosperm
zma-miR156	zma-miR156a, b, c, d, e, f, g, h	3416.73	3982.77	20459.65	5369.74
	zma-miR156j	0.15	0.08	0.07	0.63
	zma-miR156k	77.67	255.79	78.72	8.57
zma-miR159	zma-miR159a, b, f, j, k	4.98	33.31	1.15	0.1
	zma-miR159e	-	-	-	-
	zma-miR159h, i	-	-	-	-
	zma-miR159g	-	-	-	-
	zma-miR159d, c	-	-	-	-
zma-miR160	zma-miR160a, b, c, d, e, g	3.22	1.42	0.95	0.31
	zma-miR160f	-	-	-	-
zma-miR162	zma-miR162	-	0.08	-	-
zma-miR164	zma-miR164c, b, c, d, g	118.56	425.76	77.71	2.19
	zma-miR164e	18.17	8.85	3.71	212.18
	zma-miR164f	1.47	6.51	1.62	0.21
	zma-miR164h	-	-	-	-
zma-miR166	zma-miR166a	2261.6	906.37	1338.94	3871.12
	zma-miR166b, c, d, e, f, g, h, i	35.17	21.71	48	49.75
	zma-miR166k, n	103.03	16.86	14.58	4.29
	zma-miR166l, m, l	227.16	61.36	29.57	6.27
zma-miR167	zma-miR167a, b, c, d	1179.31	379.09	1541.27	7358.55
	zma-miR167e, f, g, h, j, i	159.74	143.59	83.98	319.62
zma-miR168	zma-miR168a, b	19080.41	1012.31	1578.81	33060.87
zma-miR169	zma-miR169a, b	2.93	9.02	0.34	0.1
	zma-miR169c, r	4.54	3.09	0.81	-
	zma-miR169d	-	-	-	-
	zma-miR169e	-	-	-	-
	zma-miR169f, g, h	-	-	-	-
	zma-miR169o	-	0.25	0.54	0.1
	zma-miR169l	-	-	-	-
	zma-miR169p	9.82	3.59	-	-
	zma-miR169q, m, n	-	-	-	-
	zma-miR169i, j, k	2.93	1	-	-
zma-miR171	zma-miR171a	-	-	-	-
	zma-miR171b	-	0.08	-	-
	zma-miR171c	-	-	-	-
	zma-miR171d, e, i, j	11.28	21.29	6.55	26.23
	zma-miR171f	-	-	-	-
	zma-miR171g	-	-	-	-
	zma-miR171l, m	-	-	-	-
	zma-miR171n	-	-	-	-
	zma-miR171k, h	-	-	-	0.1
zma-miR172	zma-miR172a, b, c, d	1.32	5.43	0.07	-
	zma-miR172e	6.45	7.26	0.47	-
zma-miR2118	zma-miR2118a	-	0.17	-	-
	zma-miR2118b	0.15	0.17	0.07	0.1
	zma-miR2118c	-	-	-	-
	zma-miR2118d	-	0.17	-	-
	zma-miR2118e	-	-	-	-
	zma-miR2118f	-	-	-	-
	zma-miR2118g	0.29	0.33	-	-
zma-miR2275	zma-miR2275a-3p	-	3.26	-	-
	zma-miR2275a-5p	0.29	0.58	-	-
	zma-miR2275b-5p	0.15	1.17	-	-
	zma-miR2275c, b-3p	0.44	1.42	-	-
	zma-miR2275c-5p	-	-	-	-
	zma-miR2275d-3p	-	-	-	-
	zma-miR2275d-5p	-	-	-	-
zma-miR319	zma-miR319a, b, c, d	1.03	0.33	2.5	-
zma-miR390	zma-miR390a	49.53	7.43	8.1	0.94
zma-miR393	zma-miR393a, c	-	-	-	-
	zma-miR393b	-	-	-	-
zma-miR394	zma-miR394a, b	23.59	2.84	4.12	-
zma-miR395	zma-miR395a, b.d, e, f, g, h, I, n,	-	-	-	-
	zma-miR395k	-	-	-	-
	zma-miR395o	-	-	-	-
	zma-miR395l, m	-	-	-	-
zma-miR396	zma-miR396a, b	2.34	0.75	1.35	3.97
	zma-miR396c, d	-	-	-	-
	zma-miR396e, f	-	0.33	-	-
	zma-miR396g.h	-	0.33	-	-
zma-miR397	zma-miR397a, b	0.15	0.17	0.07	-
zma-miR398	zma-miR398a, b	10.41	0.5	0.47	1.67
zma-miR399	zma-miR399a, c, h	0.73	0.17	-	-
	zma-miR399b	-	0.08	-	-
	zma-miR399d	-	0.08	-	-
	zma-miR399e, j, i	0.59	-	0.07	-
	zma-miR399f	-	-	0.07	-
	zma-miR399g	-	-	-	-
zma-miR408	zma-miR408a, b	-	-	-	-
zma-miR482	zma-miR482	-	-	-	-
zma-miR528	zma-miR528a, b	2195.36	1625.23	978.17	583.22
zma-miR529	zma-miR529	19.78	64.03	5.06	-
zma-miR827	zma-miR827	313.62	272.32	558.31	528.77
zma-miR1432	zma-miR1433	-	-	-	-

Sequencing of the four libraries showed that some miRNAs from the current miRNA database may have been mis-annotated. For example, there are two variants for miR166 in the current miRBase. First, zma-miR166b, c, d, e, h, and i are annotated as 22-nt (UCGGACCAGGCUUCAUUCCCC), while zma-miR166a is annotated as 21-nt (UCGGACCAGGCUUCAUUCCC). The 21-nt form has been sequenced 15432, 10857, 19833 and 37037 times respectively in four databases, while the 22-nt form was only sequenced 240, 260, 711 and 476 times. The 21-nt form is nearly one hundred times more abundant than that of 22-nt, therefore we concluded that zma-miR166b, c, d, e, h and i should have the same mature miRNA of 21-nt as zma-miR166a.

Consistent with the general opinion that the miRNA* degrades soon after the biogenesis of mature miRNA, the miRNA* had much less abundance than its corresponding miRNA in the sequencing dataset. Out of 167 miRNA precursors of maize in the current miRBase, 143 had miRNA* annotated. Among the annotated miRNA*, 62 of them could be found in our small RNA sequencing libraries. We also found 10 miRNA* among the remaining 25 precursors that have not been annotated before. The total sequencing abundance of miRNA* in our four libraries was about 0.7% of that of mature miRNAs. However, there were two exceptions where miRNA* had more reads than its corresponding miRNA as reported before [20]. The abundance of the originally annotated miRNA* of zma-miR396a and zma-miR396b was much higher (31, 120, 199, 59 times in four sequenced libraries) than its annotated miRNA (only 16, 9, 38, 20 in the same sequenced libraries). The same thing happened to zma-miR408, whose miRNA was sequenced less than its miRNA*. Both miRNAs had strong conservation among plant species and their target genes validated [39]. This may suggest that a small fraction of miRNA* do not degrade as fast as others.

### Novel miRNA identification and target prediction

During the miRNA biogenesis process, the pri-miRNA transcribed by RNA polymerase II is trimmed and spliced into miRNA/miRNA* duplex by Dicer-like1 (DCL1) [1]. The precise enzymatic cleavage of miRNA/miRNA* from the precursor is a key criterion that distinguishes miRNAs from diverse siRNA [36]. We observed that, for most miRNA precursors, there were few small RNA reads other than miRNA and miRNA* that mapped to the precursors. To gain an overall pattern of small RNA distribution along the miRNA precursors, we tested the percentage of small RNA reads mapped to position of mature miRNAs vs. reads mapped to other regions of the same miRNA precursors for all known maize miRNAs. The result showed that out of the 120 known miRNA precursors which had mature miRNA expressed in our four small RNA libraries, 104 (86.7%) had over 75% of the small RNA reads mapped to the exact mature miRNA/miRNA* sites or 4-nt around. Having 75% of reads mapped to the miRNA/miRNA* and its close vicinity had recently been proposed as a primary criterion for valid miRNA annotation. Our result further demonstrated that such a precise processing criterion [36] could be used as a straightforward and reliable method to identify the miRNA from the diverse small RNA data.

**Figure 2:**
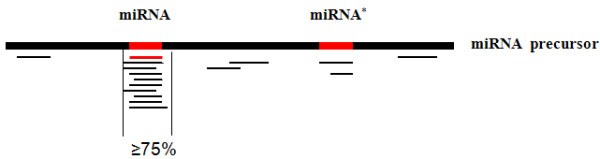
**A pictorial model for the precision of miRNA processing**.

**Figure 3:**
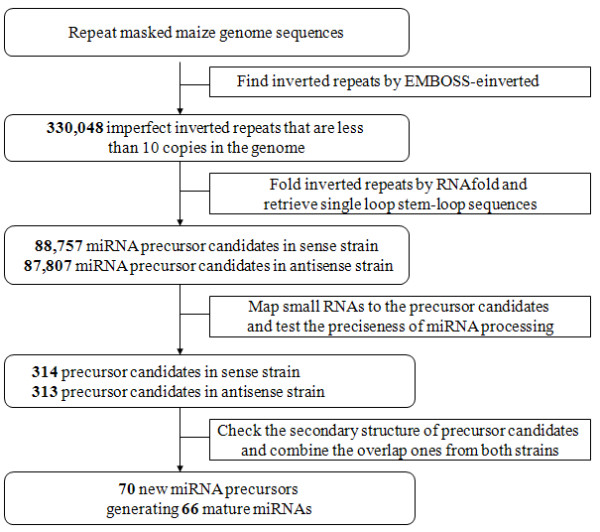
**Flowchart for miRNA prediction**.

To identify novel miRNAs using the method described above, maize genome sequences (downloaded from http://www.maizesequence.org) with known transposons masked were used to generate inverted repeat sequences. A total of 330, 048 inverted repeat sequences with a copy number of no more than 10 in the maize genome were obtained. These inverted repeat sequences were then folded by RNAfold, in both sense and antisense directions, which effectively narrowed down the candidate precursors. Candidate single loop precursors with an overall length of 80-300bp were kept in this study. We then attempted to identify novel miRNAs from our four sequenced RNA samples separately using the precise processing criterion as described in methods (Figure 2). There were 314 sense and 313 antisense RNAs that qualified as miRNA precursor candidates based on the primary criterion. Finally, the secondary structures of these candidates were carefully checked for their validity as miRNA precursors, along with their corresponding mature miRNAs (Figure 3).

There were 13 new miRNAs identified from mixed tissues I, 22 from mixed tissues II, 30 from embryo, 38 from endosperm (Table 4). All together we obtained a total of 66 unique new miRNAs. These new miRNAs could be grouped into 58 families (Table 4), given that two miRNAs with less than 4 nucleotides mismatches were grouped into one family. Sixty-two of the 66 newly identified miRNAs belonging to 54 families have not been identified before in any other organisms. Since some of the miRNAs are derived from multiple precursors, the 66 newly identified miRNAs correspond to 70 miRNA precursors. The full information and secondary structure were shown in Additional file 1 and Additional file 2.

**Table 4:** Summary of the new miRNAs

Family	miRNA	length (nt)	Sequence	Abundance (Reads per Million)			
				mixed	mixed	Embryo	endosperm
				tissue I	tissue II		
family1	miRNA1	21	CAGAAAAUCGGAGGAGAUUGA	0	0	0	1.36
family1	miRNA2	20	AGGAUACCGGAGGAGAUUGA	0	0	0.41	0
family2	miRNA3	21	UUAUAUAAGUUGGAUUAUGGU	0	0.08	0	0.94
family3	miRNA4	21	UGGAAUCAAGUGUGACAUGUU	0	0	0	1.25
family4	miRNA5	21	AUAUGGAUUGGAGGGGAUUGA	0	0	0.07	1.78
family5	miRNA6	21	ACCGGAGGGGAUUGGAGGGGC	3.96	0.5	0	0.42
family5	miRNA7	21	UUCUGGAGGGGAUUUGAGUUU	0	0	0	0.63
family6	miRNA8	20	GGGAUUGAGGGGGCUAUAAU	0	0	0.34	0.52
family6	miRNA9	21	GGGGAUUGGAGUGGCUAAAAU	0	0.08	1.35	0.84
family7	miRNA10	21	UUUGAAUGCACUAGAGCUAAU	0.29	0	0.54	5.85
family7	miRNA11	21	UUUGAAUGCACUAGAACUAAU	2.34	0.42	0.74	31.67
family8	miRNA12	21	UCCGAAUGGUGUAGAAGGAAU	0.59	1.17	0.14	6.06
family9	miRNA13	21	CUUGUGUCUUGGUUGUACGGU	0.73	0	0	0.31
family10	miRNA14	21	AGGAAUUCACUUAAUUCCCGU	0.73	0.08	0	0
family11	miRNA15	21	UGAAUUGACGAUUUUGCCCCU	0	0.75	0.07	0.42
family12	miRNA16	20	UAUCUCUACAACUAUUAAGA	0	0.08	0.81	0.1
family13	miRNA17	20	AUAUGGACGUGCAAAACACU	0	0.58	1.22	0
family14	miRNA18	21	UUUGGGGUGGAUACGUGGUCA	0	0.08	0	0.63
family15	miRNA19	21	AUGCAGAACAAUUUACAGACG	2.05	7.26	0.68	20.07
family16	miRNA20	21	AUGGUGCAUUGACUUGGUCAA	0.15	0.17	0	0.84
family17	miRNA21	20	CGACGAUCGAGAACGGCGAG	0	0	0.41	0
family18	miRNA22	20	GCCAUAGAUCUUGGCGCCGA	0	0.08	1.08	3.34
family19	miRNA23	21	UAUCUAGAAAAGCCGAAACGA	0.44	0.08	0.07	1.05
family20	miRNA24	21	AAAGCUAGAACGACUUAUAAU	0	0	0	1.25
family21	miRNA25	22	UCAGCGCCACCACGAUGACCUC	0.15	0.08	0.61	0
family22	miRNA26	22	UGAAACAAGUAUCUCGAGAGCA	25.06	0.17	0.68	100.24
family23	miRNA27	22	CAAGUGAGAGGUGGGAAUUCCC	0	0	0	0.63
family24	miRNA28	22	AAAAAGCCAGAACGAUUUAUGA	0.15	0.08	0.34	0.84
family25	miRNA29	21	UUUGGUAGUUUGAUUGGACGA	0	0	0	0.73
family26	miRNA30	20	ACCAGACUAGAGCAGCAGAU	0	15.86	0	0
family27	miRNA31	20	AUCCAUAGAGACAAAACACU	0	0.42	0.47	0.21
family28	miRNA32	21	UUUAUAAUUCGUUUGACUUUU	0.15	0.08	0	1.15
family29	miRNA33	20	AGAGACAAAAUACUGUAGAA	0	0.42	0.95	0
family30	miRNA34	21	UGGACAGGGAAAUGAAGGGGA	0.15	0	0	3.14
family31	miRNA35	21	UAGUACAUGGACCUAGAUGAC	0.59	1.5	0.47	18.81
family32	miRNA36	21	AAAUUAUAGGGCAUUUUUAUA	0	0	0.68	0.52
family33	miRNA37	20	GUUAUUUUCGGUAGCAUAAG	0	0	0.41	0.1
family34	miRNA38	21	AAAAAGAAACGGAGGGAGUAC	1.32	0.58	0.07	2.82
family35	miRNA39	21	AUACUAGGAGUGAAGGGAUCA	0.29	0	0.07	3.55
family36	miRNA40	21	UCGGGAUUGAAGGGGAUUGGA	0.73	0.08	0	2.82
family37	miRNA41	21	GGAGGGAAUUGGAGGGGCUAA	3.81	0.25	0.27	7.73
family38	miRNA42	21	UUAAUAGACCAAGACAUGCAC	0	5.26	0.07	0
family39	miRNA43	20	AUUAGUUGGCUAACUAUUAG	0	0	0.34	0
family40	miRNA44	21	AUAUGGAUUGGAGGGGAUUGA	0	0	0.07	1.78
family41	miRNA45	20	AAUUAGUCAUGGUAUGUUUA	0	0	0.34	0
family42	miRNA46	21	UGAGAGCAAGGAUACUGGAGG	0.73	0	0	0
family43	miRNA47	21	AAAUGAAACUGUAAAGGGCAU	0.29	0.67	0.54	2.72
family44	miRNA48	21	CGAAGATCTTGGGAAGATGAC	0	0	0	1.05
family45	miRNA49	21	UAGUUUGGGAACACUAAUUUC	0	0	0	0.52
family46	miRNA50	22	CUUUGACGUGGGAGAGAGGCAC	0	0	0.47	0.21
family47	miRNA51	20	AACUAAAAUGGAAUAAAAUG	0	1.59	26.53	8.15
family48	miRNA52	21	UUUUUGUGGGGGACUAUAAAC	0	0	0	0.52
family49	miRNA53	20	AACUAUUAGCUAGGAUGUUU	0	0	0.14	0.73
family50	miRNA54	21	UUCACCAUAUAAGAUUGUUGA	0	0	0	0.52
family51	miRNA55	20	GACGACCUCAGGAAGCUAUC	0	0	0.41	0
family52	miRNA56	21	UUUGGGAGCAAGUGGAAUGGA	0.15	0	0	0.52
family53	miRNA57	20	GAGACAAUUGCAUAUUUAGG	0	0.42	0.41	0.42
family54	miRNA58	20	GAAGAGGAACACAAACAGAG	0	0.5	0	0
family55	miRNA59	21	UAAGACGUUUUGACAUUUCUA	0	0.17	0.07	1.05
family56	miRNA60	21	GUGGAUUGGAUGGUAUUGAGU	0	0.17	0.2	0.52
family57	miRNA61	21	UUAGAUGGGAUACAUGAGAGG	0	0.5	0	1.67
family58	miRNA62	20	AGGGACUAAAGUUUAGUUAG	0	0.08	1.76	0.1
miR169	miRNA63	21	UAGCCAAGGAUGAGCUGCCUG	0.15	0.42	0.07	0.1
miR171	miRNA64	21	UUGAGCCGCGUCAAUAUCUCC	3.22	16.03	7.22	0.63
miR171	miRNA65	21	UUGAGCCGCGCCAAUAUCUCU	0	0.5	0.27	0
miR156	miRNA66	20	UGAUAGAAGAGAGUGAGCAC	0.88	2.25	8.91	3.14

**Figure 4:**
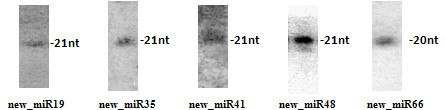
**Northern blot validation of five new miRNAs**.

From the 66 new miRNAs, 16 were sequenced in all four libraries, 17 in three, 15 in two and 18 in one library. The expressions of the 5 newly identified miRNAs were validated by Northern blot using RNAs from kernel of mixed stages (Figure 4). As additional evidence to support the annotation of some of these miRNAs, 22 of the 70 new miRNA precursors were found to have miRNA* in our sequencing data (Additional file 1).

The 54 miRNA families that were identified for the first time in maize from our sequencing effort provided an opportunity to identify conserved miRNAs that have not yet been discovered in other plant species. After searching the genomes of sorghum, rice and *Arabidopsis*, we found 17 conserved in sorghum, 14 in rice and 2 in *Arabidopsis *(Table 5).

**Table 5:** Conservation of the new miRNA

miRNA id	conservation		
	*Arabidopsis*	rice	Sorghum
miRNA3	Y		
miRNA4	Y		
miRNA5		Y	Y
miRNA6		Y	
miRNA10			Y
miRNA17			Y
miRNA22			Y
miRNA23			Y
miRNA24			Y
miRNA26		Y	
miRNA28			Y
miRNA29		Y	
miRNA32		Y	Y
miRNA35		Y	Y
miRNA40		Y	
miRNA41		Y	
miRNA42			Y
miRNA43			Y
miRNA47		Y	Y
miRNA48		Y	Y
miRNA51		Y	
miRNA53			Y
miRNA56		Y	
miRNA59			Y
miRNA60		Y	
miRNA62		Y	Y

**Figure 5:**
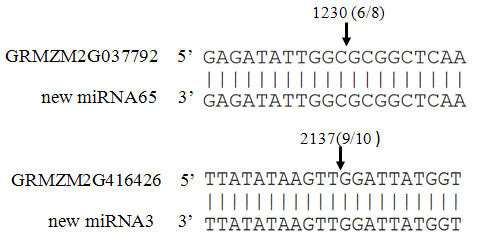
**Two validated new miRNA targets**.

As most miRNAs are near perfect complementary to their corresponding targeted mRNAs, we performed the target prediction by allowing no more than 3 mismatches between miRNA and its corresponding mRNA sequences [40]. After searching in the annotated maize filtered genes set, we found 41 targeted genes for 23 new miRNAs, 2 of which were validated by 5'RACE. GRMZM2G416426 and GRMZM2G037792 were targeted by miRNA3 and miRNA65, respectively (Figure 5). GRMZM2G416426 was predicted to be an *alcohol dehydrogenase 1 (adh1) *and GRMZM2G037792 was a GRAS transcription factor. MiRNA65 was identical to miR171a, b, c in *Arabidopsis*, which is reported to target GRAS transcriptional factor in *Arabidopsis *[41,42], suggesting that this miRNA and target pairs were conserved among dicot and monocot plants. A complete list of our predicted miRNAs and their predicted targets are shown in Additional file 3. The target gene GRMZM2G401869 of new miRNA4, was annotated to be a ribosomal protein, reported to be regulated by miR-10a in mouse [43]. MiRNA38 was predicted to target a plant specific abscisic acid (ABA) stress-induced protein (GRMZM2G027241) [44].

## Discussion

### Identification of new miRNAs according to the precision of excision from the stem-loop precursor

MiRNAs have been known to play very important post-transcriptional regulation roles throughout plant development. Identifying new miRNA is therefore a critical step towards the understanding of biological regulation. However, small RNA populations in all organisms are extremely complex; while accurate miRNAs identification is not straightforward. Thus far, the majority of reported miRNAs have been identified by "extending method" [17,19-22]. The short reads that resulted from sequencing were mapped to the known reference genome and then candidate precursors were taken by extending upstream and downstream of small map sites. The secondary structures of these extended sequences were then carefully checked for consideration as miRNA precursors. This method typically cost significant computation time, as millions or billions of small RNA sequence generated from sequencing need to be mapped to and extended in the genome individually. For any miRNA precursors, there are other small RNA sequences mapped to 4-nt around the mature miRNA, which often confuse the miRNA annotation. Lacking other supportive information, the appearance of miRNA* is regarded as an essential condition for valid miRNA annotation. However, being degraded after miRNA release, miRNA* has a much lower probability of being sequenced than that of mature miRNA. The annotation of miRNAs based on the appearance of miRNA* would often miss many true miRNAs. As the sequencing becomes relatively easily available with the development of new sequencing technology [45,46], a robust miRNAs identification system has become increasingly important. In this study, we adopted the primary criterion suggested recently by a large group of scientists in the field of plant miRNA [36]. Our method is based on an assumption that: if any sequences with stem-loop secondary structure have 75% of all small RNAs mapped onto this stem-loop fall in one distinct position (where the miRNA/miRNA* locate), then this hairpin sequences should be annotated as a miRNA precursor [36]. The advantages of our new method are apparent; it saves significant computation time, and the exact sequences of mature miRNAs for all the precursors are easy to determine. However, finding new miRNAs using this method is highly depended on the depth of small RNA sequencing, which is practical only using a next generation sequencing platform. Additionally, our method starting with the prediction of potential miRNA precursors using a very relaxed criterion, it is still possible that some precursors may have been missed, particularly for those of the multi-loop secondary structure.

Although our method relied on the precision of excision from the stem-loop precursors, as demonstrated by the small RNA sequencing data, other cleavage patterns of miRNA precursors, such as the extensive degradome sequencing in rice [47], can also be used to verify miRNA prediction. The elegant degradome sequencing results showed that most conserved miRNA precursors were cleaved precisely at the beginning or end of miRNA/miRNA* duplex.

### Additional miRNA candidates

Using this new method, we have identified 66 new miRNAs, 62 of which have not been identified before in any other organism. The discovery of these miRNAs and their targeted genes was a critical step in understanding the complex miRNA regulation network of this important crop.

According to our method, a relative high sequencing depth is required for new miRNAs identification. In our four libraries, unique small RNAs were sequenced an average of 2.6 times. Thus, we have taken 5 as the minimal abundance in the new miRNA prediction. However, some real miRNAs were not sequenced in high enough coverage and were missed. There were 50 small RNAs with a sequencing coverage lower than 5 but higher than 2. At the same time, the corresponding genomic regions of these 50 small RNA fulfill all the criteria for typical miRNA precursors; therefore, these 50 small RNAs are potential miRNA candidates (Additional file 4).

### Some miRNA precursors overlap with the protein-coding genes

**Figure 6:**
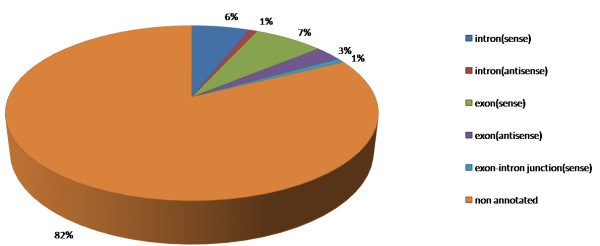
**A pie chart of the distribution of miRNA precursors in the maize genome**.

Based on the maize genome annotation release-5b downloaded from http://www.maizesequence.org/, the genome locations of the 167 known and 70 new miRNA precursors were determined. About 18% of the precursors were located within annotated protein coding genes (Figure 6). For those miRNAs that fell on genes, 10% overlapped with exons (sense and anti-sense), and 7% were located in intron regions. This result was consistent with the result reported in *P. patens *[48], where more than half of the miRNA precursors overlapped with protein coding regions.

### The small RNA population in maize is highly complicated

**Figure 7:**
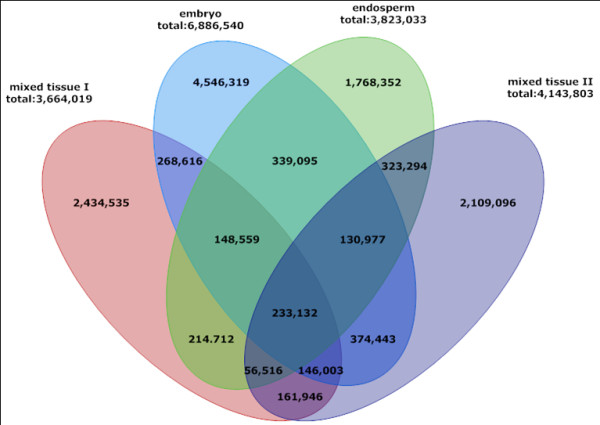
**Overlap among four sequenced small RNA libraries**.

To identify novel maize miRNA, we conducted four next generation sequencing runs for small RNAs: two mixed tissues, embryo and endosperm. Although we generated over 40 million signatures, sequences from the four databases have a limited overlap, with only 233, 132 unique sequences appeared in all four libraries and a small fraction overlapped between two libraries (Figure 7). This limited overlap indicates a very large number of small RNAs exist in maize.

We noticed that some known miRNAs had very different abundance in the four databases especially between embryo and endosperm: 30 new miRNAs were sequenced either in embryo or endosperm. For example, zma-miR168a, b and zma-miR166a had a very high abundance in the two mixed tissues and the endosperm while they could not be detected in the embryo library, which indicates that they may be endosperm specific. Although their true tissue specificity needs to be further validated through experiments, their relatively high level of expression in embryo or endosperm suggested that they could have important regulatory roles throughout embryo/endosperm development.

## Conclusion

We have implemented a novel process of identifying miRNA from small RNA sequencing data by measuring the precision of miRNA processing from precursors. Using this method, 66 novel miRNAs belonging to 54 families have been identified in maize. These newly identified miRNAs can be grouped into 58 families, of which 54 have not been identified in any other species.

## Methods

### Plant Materials and sequencing

B73 inbred was used in our study. Four separated RNA samples were sequenced. Two samples were the mixed tissues of root, stem, leaf, tassel, ear, shoot, pollen and silk. Another two samples were the tissues of endosperm and embryo. The embryo and endosperm were collected 12, 16, 20 and 24 days after pollination. For samples of mixed tissues, RNAs were extracted from 8 tissues separately by using TRIzol reagent (Invitrogen) and then mixed in equally amount for sequencing. The small RNAs of 18-28-nt in length were purified by polyacrylamide gel electrophoresis (PAGE). 3' and 5' adaptors were added for RT-PCR amplification and PCR products were subjected to sequencing. Low quality reads and the adaptor sequences were removed before further analysis.

### Data analysis

All the reads generated from sequencing were mapped to the maize genome sequences (release-3b.50, http://www.maizesequence.org/. Reads that could not perfectly map to the genome were excluded. RepeatMasker http://www.repeatmasker.org) was used to filter the reads from repeat elements. Known non-coding RNAs including tRNA, rRNA, snoRNA, snRNA and other non-coding RNA sequences were annotated by comparing the reads with the Rfam database http://www.sanger.ac.uk/Software/Rfam/ using BLAST (identity ≥ 80%). The known mature miRNA sequences and precursors were downloaded from miRBase (Release 17.0; http://microrna.sanger.ac.uk/sequences/. Sequences matched to the known miRNA precursors were excluded in the miRNA identification pipeline.

### Novel miRNAs identification and target prediction

The maize genome sequences masked with the MIPs repeat were downloaded from B73 genome project [49](release-3b.50, http://www.maizesequence.org/. Inverted repeat sequences were extracted by EMBOSS-einverted [50] from the masked genome sequences using parameters "-gap = 20, -threshold = 60, -match = 5, -mismatch = -4, -maxrepeat = 400". As we noticed that a sequence can have different secondary structures in the sense and antisense direction when calculated by RNAfold, the inverted repeat sequences were folded in both directions to retrieve stem-loop sequences with single loops as the candidate miRNA precursors.

We then attempted to identify novel miRNAs from four sequenced RNA samples separately. For each sequenced RNA samples, short reads were first mapped to all the candidate precursors. The precursors that fulfilled the following conditions were selected for further analysis: 1) precursor had at least one unique small RNA of 20~22-nt mapped on it, 2) the unique small RNA had at least five identical reads in a sequencing library, 3) the genomic sequences of the unique small RNA are less than 20 copies in B73 genome. For each selected precursor, a particular position that had the highest number of identical 20-22-nt small RNAs mapped to it was regarded as the potential mature miRNA.

Finally, distributions of reads for all the mapped small RNAs for the selected precursors were checked. If the number of small RNAs mapped around the potential mature miRNA (including 4-nt upstream and downstream) account for 75% of all the reads mapped to the precursors, then the candidate precursor was regarded as a true miRNA precursor, while the most abundant small RNA mapped on it was regarded as the mature miRNA.

After screening by the primary criteria, the secondary structures of the precursors were predicted again by RNAfold [51] using additional parameters. The secondary structures of the inverted repeat should satisfy the following: the MFEI [32](minimum free energy calculated by RNAfold divided by the sequence length) should ≤-0.15; the miRNA candidates should be on the stem of the stem-loop sequences; the candidate miRNA and miRNA* should have no more than 5 mismatches. Inverted repeat sequences that passed all the filters were regarded as our new miRNA precursors, and their corresponding mature miRNAs were the small RNA with the largest abundance among the ones mapped to them.

To find additional evidences for the newly identified miRNAs, the expression of the precursors were tested by BLAST analysis with existing expressed sequence tag (EST) database. We also tried to find the miRNA* in our four small RNA libraries as additional evidence for the annotation of new miRNAs.

To find conserved miRNA in *Arabidopsis*, rice and sorghum for newly identified miRNAs in maize, genome sequences of *Arabidopsis*, rice and sorghum http://www.arabidopsis.org/, http://www.tigr.org, http://www.phytozome.net/ were downloaded. If the new miRNAs have conserved sequences of no more than 4 mismatches in the genome, we extend the corresponding sequences for further analysis. Two extensions were made: one upstream 30-nt and downstream 300nt, the other upstream 300nt and downstream 30-nt as putative precursors, for the reason that mature miRNA are on the 3' or 5' stem of its precursor. Then the putative miRNA precursors' secondary structures were predicted by RNAfold. If the secondary structure fulfilled the criterion for miRNA precursors, we considered the miRNA conserved in the genome.

The maize annotated coding sequences and Go annotation were downloaded from B73 genome project [49]http://www.maizesequence.org/. release-5b). Because most miRNAs were near perfect matches to their corresponding target mRNA, we identify the miRNA target using BLAST with no more than 3 mismatches between miRNA and target sequences.

### New miRNA validation by northern blot

The RNA gel blot hybridization was performed as described previously [52]. Total RNA was extracted using RNA pure plant kit (TIANGEN of Beijing, DP437). The low-molecular-weight (LMW) RNA was detected by 15% polyacrylamide gels, blotted onto Hybond N+ membrane (Amersham BioSciences) using the transferring machine (Bio-Rad Laboratories, Mini Trans-Blot) for 1.5 hours at 200 mA, and UV cross-linked. Blot probes for specific small RNAs were labeled using γ-^32^P-dATP by T4 polynucleotide kinase (NEB, M0201). Blots were prehybridized and hybridized at 37°C for 7 and 24 hours respectively using perfectHyb plus hybridization buffer (Sigma, H7033). Blots were washed at 50°C first with 2 × SSC/0.2% SDS for 15 minutes one time, the second wash was carried out using 1 × SSC/0.1% SDS for 10 minutes, then repeated.

### miRNA target validation by 5' RACE

Total RNA was extracted from maize (B73 inbreed) 14 days seedlings, then treated with RQ1 DNase I (Promega) for half an hour. cDNA templates were prepared by following the instruction of SMART RACE kit (Clontech). Two gene-specific primers (GSP1 and NGSP1) were designed by Primer Premier 5.0 software. These two primers were used for two rounds of PCR. Nested PCR products were analyzed on an agarose gel. Positive PCR products were cloned into pEASY-T1(TransGen) vector by use of pEASY-T1 Cloning Kit(TransGen). Each target sequence was confirmed by at least 7 clones.

## Authors' contributions

JL designed the research. YJ did the data analysis. WS and MZ performed the sample preparation and experimental validation. JL and YJ wrote the paper. All the authors have read and approved the final manuscript.

## Supplementary Material

Additional file 1 **new miRNA precursors**. new miRNA precursors. the location of the new miRNA precursors in maize genome Click here for file

Additional file 2 **secondary structure of the newly identified miRNA precursors**. the secondary structure of the newly identified miRNA precursors. the secondary structure of the newly identified miRNA precursors. Click here for file

Additional file 3 **the target gene of new miRNA1**. the target gene of new miRNA. the target gene and annotation of new miRNAs Click here for file

Additional file 4 **candidate miRNA precursors**. candidate miRNA precursors. the location of the candidate miRNA precursors in maize genome Click here for file
