# Supplementary material for: Identification of novel maize miRNAs by measuring the precision of precursor processing
Source: BMC Plant Biol. 2011 Oct 20;11:141. doi: 10.1186/1471-2229-11-141 (PMC3214924; doi:10.1186/1471-2229-11-141)
Supplement: Additional file 2 — secondary structure of the newly identified miRNA precursors. the secondary structure of the newly identified miRNA precursors. the secondary structure of the newly identified miRNA precursors. [file 1471-2229-11-141-S2.PDF]

```
>miRNA1
           C                   A  AA     U
AGUUUGGGAGC AGAAAAUCGGAGGAGAUUG GA  GAGAU U
UCAAACCUUCG UCUUUUGGUUUCCUCUAAC CU  CUCUG U
           A                   -  --     U

>miRNA2
     -    CAA             -      -C   -C           A
AGGGU UUGG   AGGAGAUUGGAGG GCUAAA  UCU  CUUGCUAUUUA U
UCCUA GGCC   UCCUCUAACUUUC CGAUUU  AGA  GAAUGAUAAGU U
     U    ---             U      U-   A-           U

>miRNA3
   G               CC                         U      -C-  AUA-      CAUCAC--    UAAU-            ACUC    CU
AGG UAUGUUUGUUUCUCU  UAAAUUAUAUAAGUUGGAUUAUGGU GAAGGA   GG    GUAAAA        UUAG     UAUAAAUAAGCU    AAUG  A
UCC AUACAAACAGGGGGA  AUUUAAUGUAUUCAACCUAAUGCCA CUUUCU   UC    CAUUUU        AAUC     AUAUUUAUUCGA    UUAU  A
   A               AA                         -      CAU  -ACG      -AUAAUAA    --CUC            -CU-    UU

>miRNA4
     AC             G    AC   -  -                  C-    A-   A-  A-     AA                      A  A              G-     CUGUCUU         U
UUUGU  CUCCAAAUAAUGU CUUC  AUU UU UCAUUCGUAACAUUGGCU  CGCA  AAG  AG  ACUCA  ACAUGUCACACUUGAUUUCAAU CC UUUUGGACAUUUUU  CCCUU       UUGUGUGUG A
AAACA  GAGGUUUGUUACA GAAG  UAA AA GGUAAGUAUUGUAACUGA  GUGU  UUU  UC  UGAGU  UGUACAGUGUGAACUAAGGUUA GG GAAACCUGUAAAAA  GGGAA       AACACACAU C
     CU             A    CA   C  C                  -A    -C   -A  -C     --                      C  C              AA     -------         G

>miRNA5
    C    C        A A          A      GGGGG
AGGG UUGU UGGUUAGA G GGAUUGGAGG GAUUGA     G
UCCC AACA ACCAAUCU U CCUAACCUCC CUAACU     A
    A    A        A A          C      AGUAA


>miRNA6
    UC            -  AUU   -            AAAUA   A A  A    UUCCUCUU
ACGC  AGGGCUAGUUUG GA   CCA AAAACCGGAGGG     GAG G CU GAAU        A
UGCG  UCCUGAUCAAAC CU   GGU UUUUGGCCUCCC     CUC C GA CUUA        |
    CA            U  -C-   G            CUAAC   C C  C    UUUAACUU
```

```
>miRNA7
                               U        U          A    GC
GGGCUAGUUUGGAAACUCAAAUCCCUUCCGG AUUGGAGG GAUUGAGGGG GAAU  A
CUCGAUCAAACCUUUGAGUUUAGGGGAGGUC UAAUCUCC CUAACUCCUU UUUA  A
                               U        U          C    AU
```

```
>miRNA8
               C   U A    A         A     C  U                A
GGGCUUGUUUGGGAG AAG G AAUG AGGGGAUUG GGGGG UA AAUCCCCCACUAUUUA A
CCCGAACAAACCCUC UUC C UUAU UCCCCUAAC UCCCC AU UUAGGGGGUGAUAAGU A
               A   C A    C         C     A  C                U
```

```
>miRNA9
     -             G-        GCUAAUAAGG
AAACG GAGGGGAUUGGAG  GGCUAAGG          A
UUUGU CUCCCCUAACCUC  CCGAUUUU          G
     A             -A        AUUUAGGGGA
```

```
>miRNA10_1
     C  A                   -A           -CUUUU  AU
UUUAG CA CUAACUAUUAGUUCUAGUG  AUUCAAACACU      AG  A
AAAUC GU GAUUGAUAAUCGAGAUCAC  UAAGUUUGUGG      UC  U
     A  -                   G-           UCCAU-  AA
```

```
>miRNA10_2
     UUU     CCGG   U    U   A        A C   C    A          G  C      A  AUUC     U      -UG  GCAA
AGCUA   GUUAG    CUA UUUU UUA CAAUUUUU G CAA UAAU AUUAGCUCUA UG AUUCAA CA    CUUAG CAAAUU   AC
UCGAU   CAAUC    GAU AAGG GAU GUUAAAAA C GUU AUUG UAAUCGAGAU GC UAAGUU GU    GAAUU GUUUGG   UG
     UAU     AAUA   U    U   C        A A   U    A          G  A      A  ACGA     -      UG-  ----
```

```
>miRNA11_1
   A                            CAACACA                        A                      UUCCGUUUGACAU  -U     -C  U  A
UAG UAGUUAUUUGUUAGCUAGCUAAUUCUAC       UUUUAGCCAACUAACUGUUAGUU UAGUGCAUUCAAACACCCCUUA             AG  AAAAG  AG AC C
AUC AUCAAUAAAUAAUCGAUCGAUUAAGGUG       AAAAUCGGUUGAUUGAUAAUCAA AUCACGUAAGUUUGUGGGGGAU             UU  UUUUC  UC UG  |
   C                            AUUAUUA                        G                      UAAAAUCUUCCU-  U-     CU  U  A
```

```
>miRNA11_2
       C               CC C       U  UUUUUUUAU
AUUAGUU UAGUGCAUUCAAACA  C UUAAGUCA AG         C
UAAUCGA AUCACGUAAGUUUGU  G GAUUCAGU UC         U
       A               AU A       C  UAAUUUCUU
```

```
>miRNA12
    A G     A   CUA  U    G    U    C        C  C      AUCCUAAU  -                 A      A   -     CC   U    -G              ------ACA  AGAAA
AAUU U UAUAU CCA   CA UUUU UCUA UAC UUAUUUGC AU GUAAAU        UC CUUCUACACCAUUCGGAU CAAUUU UUA CUUUU  AUG CAUC  UCAGUGGCAUAGAA         UA     A
UUAA G AUAUA GGU   GU AAAA AGAU AUG AAUAAACG UA CAUUUA        AG GAAGAUGUGGUGAGUCUA GUUAAA AAU GGAAA  UAC GUAG  AGUCACCGUGUCUU         UG     U
    G G     C   AAC  U    A    C    A        A  C      CAUAACU-  A                 C      G   A     -A   -    A-              UCUUAAUCC  UAUAU
```

```
>miRNA13
             AU        AACCA     -A        -A  -A         -A   -G      -UA  -AG     -C  A      -AU      ------A---------G
GGGAACAUAUCAG  GCAACUGU     UUGGU  UAACCGU  CA  CCAAGAC  CAA  GAUGAA   UG   AGGAC  UU UAAAAA   AUCCAU                 G
CCCUUGUAUAGUC  UGUUGGCA     AAUCA  AUUGGUA  GU  GGUUCUG  GUU  CUACUU   AC   UCCUG  AA AUUUUU   UAAGAA                 A
             CU        AAAA-     C-        C-  A-        G-   A-      UC-  CA-     C-  -      A--      AAAAAC(15bp loop)G
```

```
>miRNA14
                                  C              U    C         CUU           UUU
ACGUAGAUAUUCUUUUUUCGUGUAUAUAAUCUGC CAUUGUUUAUAUAC CUGC GGAAUUCGC   AUUCCCGUCGG   C
UGCAUCUAUAAGAAAAAAGCACAUAUAUUAGAUG GUAACAAAUAUAUG GAUG CCUUAAGUG   UAAGGGCAGCC   |
                                  A              U    U         AAU           UGA
```

```
>miRNA15
      A  A                  A   U     -         C                  C    A        C    UUAGU       --CU                -C      -A        UAAA
ACUUAU UG CCCUUCCAUUAGUUUUGG GCU AAAUG UGUUAAGUC AGGGUGAAUUGACGAUUUUG CCCU GGUGGAAA UGAA     GCAGAGUU    GUUUGUCUAUGUUUAG  UUUGAG  AAUAUUUU    C
UGAAUA AC GGGAAGGUGAUCAAAACC UGA UUUGC ACAAUUCAG UCCCACUUAACUGCUAAAAC GGGA UCACUUUU ACUU     UGUCUCGG    CAAGCAGAUACGAGUU  AAACUC  UUAUAAAG    C
      C  C                  C   -     C         U                  U    C        U    -----       AU--                A-      C-        UGUA
```

```
>miRNA16
        U--      U     AUAA   --UC A    CCUACU      UA   UAUAUC
ACUCUUAA   AGUUG AGAG    GUA    C AUAA      UAUAGA  GUU      A
UGGGAAUU   UCAAC UCUC    CAU    U UAUU      UAUCUA  UAA      C
        UAU      -     ----   UU-G A    -----A      A-   CUCACC
```

```
>miRNA17
     A     -G     GUACCU   AUUA  C  U    A
AAUAG UGUUU  GUAUG      UUU     G UG CUCC G
UUAUC ACAAA  CGUGC      GUA     G AC GAGG |
     -     A-     ----AG   UAC-  U  -    C

>miRNA18
           A   C         C   A         UCAGG    -GU   UGU
UUGUUUGGAUG CCA GUAUCCACC CAA CCAUGUGUG     GUGG   UGA   G
GACAAACCUAC GGU CAUAGGUGG GUU GGUACACAC     CGUC   AUU   G
           G   A         A   A         CUUA-    AA-   UAA

>miRNA19
                 A           C-             A-   C-      A-    G-             CG-      A-        AUUUCUCA-   GCA
UGCCUUUUAUCCAAACA CAGUUUCUAAA  GUAAAAUAGAACA  UAG  AUUCUU  UGCA  AACAAUUUACAGA   GAAAUG  UUUGUACU         GGU   A
ACGGAAAAUAGGUUUGU GUCAAAGGUUU  UAUUUUAUUUUGU  AUC  UAAGAA  ACGU  UUGUUGAAUGUCU   CUUUAC  AAACGUGG         CCA   A
                 C           UA             -C   -A      -C    -G             -AG      -C        -ACUCUUUA   ACC

>miRNA20
      U              CCCC   --AUU  -A             -C           --CC  -C   -U  U
UUUUCA CUUUGCAUCAUUGC    CUG     UG  CCAAGUCAAUGCA  CAUUGCCCCUG    UU  GCC  UC U
AAGAGU GAAACGUGGUAACG    GAU     AC  GGUUCAGUUACGU  GUAACGGGGAU    AA  CGG  AG |
      U              AC--   GAU--  A-             A-           CA--  A-   U-  U

>miRNA21
     -        --     -   UAG  GA
 UCU GUUGUUUU  GUCGU GUC   GC   G
 AGA CGGCAAGA  UAGCA CAGUGGUG   G
    G       GC      G        AG

>miRNA22
  -    -      G              CCC-    CA  AUU
GG GCCA AGAUCU UGGCGCCGACCCCC    CAGA  GG   U
CC CGGU UCUAGA ACCGCGGCUGGGGG    GUCU  CC   |
  G    A      -              -ACA    --  ACU
```

```
>miRNA23
     C             A   -A  -A               -U    -U   -UUUU -CG      A GUACAC
AUUCU UUUGUUUCAAAUU UAA  UC  UUUCGGCUUUUCUAG  UACA  AGC     G   AUGUAU C      U
UGAGG AGGCAAAGUUUAA AUU  AG  AAAGCCGAAAAGAUC  AUGU  UCG     C   UACGUA G      C
     U             -   C-  C-               U-    U-   CCUU- AA-      - AUCUAU

>miRNA24
         C                            A A       UAAAA           A  C GAA
UCCCUCCAU CUGAAUUAUAAGUCGUUCUAGCUUUUUU G UACAUAG     GUUAUGUAUCU GA A   G
AGGGAGGUA GGUUUAGUAUUCAGCAAGAUCGAAAAGA C AUGUAUC     CAAUACGUAGA CU U   U
         A                            C C       UAAAA           C  A AUG

>miRNA25
   C      -  AA    -  A          CCCCUCAC U  UA   U      GA C   -AUCAU  UU
AAG GAGUGG GA  GCCU AG CGCCGCCAUG        A GC  CGC CGUGG   G GC       CA  G
UUC CUUACC CU  CGGA UC GCGGUGGUGC        C GG  GCG  CGCCC  C U G      GU  C
   C      G  -G    G  -          ---U--A- U  A-   G      GC G   AUU---  UA

>miRNA26
    A    GUAGA    -    AGA---------        -    A
AUGA UAAA     UGCU CUCG            UACUUGUU UC  |
UACU AUUU     ACGA GAGU            AAUGAAACAAG  |
    A    AACAC    A    -GAAAUCCCAAG            G

>miRNA27
     UCAG    A         A     -  GA         A   C       U      -CACCUUUUU      CU
AUCCC    AUCC AUUUGAGUG CCCAA GU  GAGGUGGGA UUC CUCAUCC CUUCUC          ACUGGA  A
UAGGG    UAGG UAGGUUCAC GGGUU CA  UUCCACCCU AAG GAGUAGG GGAGAG          UGACUU  |
     UAAG    C         C     U  -A         C   A       -      CAU-------      AG

>miRNA28
   AC-    AA    AA      C     G    C    A       G      U  -A      -C  -C  UUACAG
CUC   GAUU  GAAC  CUCCCU CGUUC AAAU AUAA UCGUUCU GCUUUU CU  GAUACA  AA  CU      U
GAG   UUAA  UUUG  GAGGGA GUAAG UUUA UAUU AGUAAGA CGAAAA GA  CUAUGU  UU  GA      |
   AUU    -A    AC      U     G    A    C       G      -  A-      A-  A-  UCUAUA

>miRNA29
         A   GUAAUA   -    A               -C -A                 -A                -AA-        A-                      AUU-                 UAU      -UC    ---UU   --UA   C
CAAAAACUG AGU      CAA CUUU UUGUGCAAUUGUCGU  C  AUCAAACUACCAAAAGU  AUGAAAACUUCUAAAA    UUAUAGAU  UAAUUUAUUGUUUACUUUACCA    UAUCAAAUUUUAGGUUC   GAAUUU   UCUU     UUA    GCU U
GUUUUUGAC UCA      GUU GAAG AACACGUUAGUGGUA  G  UAGUUUGAUGGUUUUCA  UAUUUUUGAAGAUUUU    AAUAUCUA  AUUAAAUAAUAAAUGAGAUGGU    AUAGUUUAAAAUUCAAG   CUUAAG   AGGA     AAU    UGA U
         C   AGUCA-   C    -               U- C-                 G-                CUUA        -C                      -CAU                 ---      U--    UAC--   UC--   A
```

```
>miRNA30
    A      CAC    A   -        -C  A AAU
UGUA GGUUUG   UUGU CUG UGCUCUAG  CU G   U
ACGU CCGGAU   AAUA GAC ACGAGAUC  GA C   U
    A      AC-    -   G        A-  - CAU
```

```
>miRNA31
  G     -    G-                                                                                      CUA-                  U-               G-    A-          -----UAGCUGCAUCAGUCU
AU GUUAU UUGG  GUCGCCAAUUGAGGCGCAGUGUAACCGGUCUACUUUAGACCUUUCUUUCUAGGUUGUUCGCUUCGGAUUAAAGCUAGUUGUUCAGA    AUGCAGCUGCAAUCCAUA  AGACAAAACACUAUA  AAAC  GUGGAAGCUU                    A
UA UAAUA AACC  CAGCGGUUAACUCCGCGUCACAUUGGCCAGAUGAAAUCUGGAAAGAAAGAUCCAACAAGCGAAGCCUAAUUUCGAUUGACAAGUCU    UACGUUGACGUUAGGUAU  UCUGUUUUGUGAUAU  UUUG  UACCUUUGGA                    |
  G     U    -A                                                                                      -UUC                  -C               -G    -C          AGCCUAAUUUCGAUCGACAA
```

```
>miRNA32
            C                 GAC      -ACAACU  -U    -C      -U       -U      G   --UAAU   U----AUUUAU  A  -AUU        ---GUUU GC    U
ACUCCCUCUGUU CAAUUUAUAAUUCGUUU   UUUUUU       AA  UUGA  UAGCUU  UCUUAUU  AAAAAU UCA      UAU           UC U     GUCAUAUU       A  AUA A
UGAGGGAGACAA GUUAAAUAUUAAGCAAA   AAAAAA       UU  AACU  GUCGAG  AGAAUAA  UUUUUA AGU      UAA           AG U     CGGUAUGA       C  UAU |
            A                 AU-      GAUUU--  U-    A-      C-       U-      -   UU---A   ACUUUUU----  A  UU--        AUUU--- A-    A
```

```
>miRNA33
   C                      U  CAAA    GA    -  A
CUU UACAGUAUUUUGUCUCUAUGGA UA    UACA  GCAG CC A
GAA AUGUUAUAAAACAGAGAUACUU AU    AUGU  UGUC GG A
   U                      U  AUCG    --    A  U
```

```
>miRNA34
      G        A    C          CA   AA
UGGUUU GUGGACAG GAAA GAAGGGGAUC  UGG  G
ACCAAA CACCUGUC CUUU CUUCCCCUAG  ACC  C
      A        C    A          CA   CC
```

```
>miRNA35
         A                    ---    UU-  A-   CA---    A    UU
CUAUUUCGG UGUGUCAUCUAGGUCCAUGU   ACUA   CA  GGC     CAUU CCCA  G
GAUAAGGCC ACACAGUAGAUCCAGGUACG   UGAU   GU  CCG     GUGG GGGU  G
         -                    UAU    --U  -G   -CAAG    -    CA
```

```
>miRNA36
          -----            UAAG CA
UUUAAAUUAU     AGGGCAUUUUUA    U  C
AGAUUUAAUG     UCUCGUAGAGGU    U  |
          AUUUC            --UG UA

>miRNA37
      C-  C-  CCA-  C-           U-       C-  C-        A
UUUUCG  GG  CA    AC  AUUGAAAAUAG  CCUUAUG  UA  CGAAAAUA C
AAAAGC  CC  GU    UG  UAGCUUUUAUU  GGAAUAC  GU  GCUUUUAU |
      CA  -A  -UUC  -A           -U       -U  -A        U

>miRNA38
      GU     --CAACA  A     - -C     ---UGU         --CC    --CU     ---CAA      UCCAACAGCCA
UGGGUG  UUGUU       GU CAAUC C  AGUGC      GUACUCCCU    GUUU    UUUUA      GUCGUU           A
ACCCAU  GACGG       CA GUUAG G  UUACG      UAUGAGGGA    CAAA    AAAAU      CAGCGG           U
      --     CUUA---  -     U U-     CUU---         AA--    AC--     CUA---      CUAUAAAUAUC

>miRNA39
                    G    CU  C  A            U                  C  A       A  G       A    CC
GAGCUUUUGUCAAACUUUCU UCUG  GU UA CUCAUAGUCUAC CUAGAUGAUCCCUUCACU CU GUAUACU UU AUAAAUU UUUC  C
CUCGAAAACGGUUUGAAAGA GGAC  CA AU GAGUAUCAGAUG GAUCUACUAGGGAAGUGA GA CAUAUGA AA UAUUUAA AAAG  G
                    G    AU  A  A            C                  A  C       C  A       -    AA

>miRNA40
       C         C    -AUCC                   -A  -A  -U      GAA
AGGGCUA UUUGGGAAC UCAA     CCUUCGGGAUUGGAGGGGA  UG  GG  GGAAAU  A
UCCCGAU AAACCUUUG AGUU     GGAAGCCCUAACUUCCCCU  AC  UC  CCUUUA  C
       U         -    AAAU-                   A-  C-  U-      AUC

>miRNA41_1
    U  UUUA    UC  --GA        -A    -GAG            --AA            -A       -A         A
CAUG UG    ACUA  AG    CUAGUUUG  GAGC    AAAACCGGAGGG    UUGGAGGGGCUA  AUUUCCC  UCUUAUUCA A
GUGU AC    UGAU  UC    GAUCAAAC  CUCG    UUUUGGCCUCCC    AACCUCCCCGAU  UAAAGGG  AGAAUAAGU A
    U  UCAG    --  AC--        C-    GUA-            CC--            C-       A-         U
```

```
>miRNA41_2
      C       A    CA     C A        UA                          A
AAGGGC AGUUUGG AGUC  AAACC G AGGGGAUU  AGGGGCUAAAAUCCCCUUUUUAUUCA A
UUCCCG UCAACC UCGG  UUUGG C UCCCUUAA  UCCCCGAUUUUAGGGGAGGAAUAAGU A
      A       C    AG     A C        CC                          U

>miRNA42;21;15;35
                 C          C      GUC
UGGUUUGAUUCUGUGUG AUGUCUUGGU UAUUAA   A
ACUAAACUAAGACACGC UACAGAACCA AUAAUU   U
                 A          A      GAU

>miRNA43
   -   A-    CAA-             UGACUCAACCC
CUC ACA  CUAA    UUAGUUAAUUAAU           U
GAG UGU  GAUU    AAUCGGUUGAUUA           |
   A   -C    -AUC             UUUAAUAAUCG

>miRNA44
                 UAU                 UCAUU
GGGUUUGUUUGGUUAGA   GGAUUGGAGGGGAUUGA     U
CCCGAACAGACCAAUCU   CCUAACCUCCUCUAACU     C
                 UCU                 CCCCC

>miRNA45
       UG    G       GA---    U    -G-----GCC  GUUUG AUCCUCUGGUUAAUU
UUUGGAU  ACUA GACUAGU     UUAG CUAA          UU                     U
AAAUUUG  UGGU CUGAUUA     GAUC GAUU          CG                     U
       UA    A       ACAAC    -    UAUAAAUAAC  AUUAA CAAUCAAUGACCGUU

>miRNA46
    A                G                              CA      A     AG   UAAAA
CGGU GGUGAUCGAUUGUAUA CAUUUAGGGCUUGUUUGAGAGCAAGGAUAC  GAGGGG UUGAA  GGC     U
GUUA CCACUAGCUAACAUAU GUAAAUCCCGAACAAACUCUCGUUCCUAUG  CUCCUU AACUU  UCG     U
    A                A                              AC      A     A-   UUCUC
```

```
>miRNA47
                  A                             A   C          A    --C AGAUAGCGCACAUC
ACAACUAUGCCCUUUGCA UUUCAUUUUAAAAAAUGGACCCUUGGUUC GCG CGUCAUCAUU GCGC   G              A
UGUUGAUACGGGAAAUGU AAAGUAAAGUUUUUUACCUGGGAACCGAG CGC GCAGUAGUAA CGCG   G              U
                  C                             C   A          C    ACA UAGUAAGUUGUUUA

>miRNA48
   C   U      --    --     ---   CAA---   C
UAG UUC CGGAGU  CAGU  CAUCU   UCC      GAU U
GUC AAG GUUUCG  GUCG  GUAGG   AGG      CUG U
   U   -      AC    CU     UAC   CCAUCG   C

>miRNA49
                          -       UUUC      CC
GGGCUAGUUUGGGAACACUAAUUUUC AUGGGAU    AUUUUC  A
CCCGAUCAAACCCUUGUGAUUAAAGG UACCCUA    UAAAAG  A
                          G       -U--      AA

>miRNA50
            -------G-     GA   A   AUC       CC    CGUGC
AUCCUUUGACGU         GGAGA  GGC  CU    AUGUGG  CCCG     C
UAGGAGACUGCA         CCUUC  CCC GUG   GUACAC   GGGG     |
            UAACGUACC     AU   A   -AC       CC    ---CA

>miRNA51
            C                      AC          A       UCCA      C                   U
GGUGUUUGGUUU UAGGGACUAAUGUUUAGUCCCU  AUUUUAUUCC UUUUAGU    AAAUUG UAAAUAUAGAAACUAAAAU U
CCACAAACCAAA AUCCCUGAUUACAAAUCAGGGA  UAAAAUAAGG AAAAUCA    UUUAAC AUUUAUAUCUUUGAUUUUA |
            C                      AU          A       UAUA      A                   U

>miRNA52
    AUA       C            A       U    C   UAUAAACUUACUAUU       GU
UAGG   UUUAUAG CCCUCACAGAAA AAAGAUA AUCC ACC               AGAUAAC  U
GUCC   AAAUAUC GGGGGUGUUUUU UUUCUAU UAGG UGG               UUUAUUG  U
    CAC       A            C       C    A   UCGUCGUC-------       AU
```

```
>miRNA53;
      GA-       CUA--       A  UA-    A
CAAAUA   UAGCUAA     UUAACUA UU   CUA A
GUUUGU   AUCGAUU     AGUUGAU AA   GAU A
      AGG       -AUCA       -  -UC    A

>miRNA54
                 C-                 A-  CAA-         A-      A-            C-    A-  AGAAA     AC
UGUUCAUAUGUUUUUUU  CUCUGGUGUUAACUUAA  AU    CAAUCUUAU  UGGUGA  CAUCAAUGUCUA  AUUU  UU     UUUUU  U
ACAAGUAUACAAAAAAA  GAGACCACAAUUGAAUU  UG    GUUAGAAUA  ACUACU  GUAGUUAUAGAU  UAAA  AG     AAGAG  |
                 AC                 -C  -AUC         -C      -C            -A    -A  -----     GC

>miRNA55
        A C    GA--             AAUA
UUUCGACG C UCAG    AGCUAUCGGACGU    U
AAGGCUGU G AGUC    UCGAUAGCCUGUA    |
        C A    ACGG             CAUA

>miRNA56
        A         A    U   U                    CC            A    GGGGAU     GUCU
CUUGUUUG GAGCAAGUG AAUG AGG GAUUGAGUGGGCUAGAAUCC  UAUUAUUCAAGA UGGA      UGAGG    A
GAACAAAC CUCGUUCAC UUAC UCC CUAACUUACCCGAUCUUAGG  GUGGUAAGUUUU ACUU      AUUCC    C
        C         C    C   C                    CC            A    AUU---     AUAA

>miRNA57
CC    -U  -G              -C  --AACU      G
  UAAA  AU  CAAUUGUCUCCUUU  CU      UGGUG C
  GUUU  UA  GUUAAUAGAGGGAA  GA      ACUAU G
--    U-  G-              C-  AUUU--      C

>miRNA58
    C   ------      ACACAA         -  - CAUACUGUAAAUAACGCCCCCACAGAGAAG
CCUU GCA      GGAACA       CAAACAGG CC A                              C
GGAG GCG      UCCUUG       UGUUUGUC CG G                              A
    A   AACUUC      -------        U  A UACAAAAAAUAAAACGAGACGUAAUAGAUG
```

```
>miRNA59
       C     CC    C               A-         UUCUA-       A-     AC         C
UACUUUC CUGUU  AAAU AUAAGACGUUUUGAC  UUUCUAUAU      GCUUUUG  UAUGU  UUAGGUAUA A
AUGAGGG GAUAA  UUUA UAUUUUGUAAAACUG  AAAGAUAUA      UGAGAAC  AUACA  AAUCUGUAU |
       A     AA    A               AA         -UGUAA       -G     -C         C

>miRNA59
   C    A    G                  U U              -         CGCAA---       U
ACU CCUC GUUU AAAUUAUAAGGUGUUUUG C UUUCUAGGUUCAUA CUUUUGCUA        AGAUGUA G
UGA GGAG UAGG UUUAAUAUUCUGCAAAAC G AAAGAUCUAAGUAU GAAAAUGAU        UCUAUAU |
   A    G    G                  U U              C         -ACAUAGA       G

>miRNA60
            GA-   ---       UC--  U  AA--  CUAUAAAU
CUAUGUGGAUUG   UGG   UAUUGAG    GG UU    AU        C
GAUAUACCUAAC   ACC   AUAACUC    CU AA    UG        |
            AUC   CUA       --UA  U  -AAC  AACGAUAU

>miRNA61
    AUCC  U-CC      ---AGACUA GU         -G     AA           -A   UC        -A     CC          A
CUCA    CC    UUCCAA         A  GCUGAUUUG  UAACA  AGAAUUGGAUG  GA   CAUGAGAG  AAAUC  UUGUUAUUUA U
GAGU    GA    AGGGUU         C  CGACUAAAC  UUGUU  UCUUAAUCUAC   UA  GUACUCUC  UUAGG  AAUGAUAAAU U
    ---G  AU--      AAA-U---- C-         AA     A-           CC   U-        CU     U-          U

>miRNA62
        CGUUUUAUUCCUU  GUCC         AG       A
UAAGACCA             UA    AGGGACUAA  UUUAGUU G
AUUUUGGU             CA    UCCUGAUUU  AAAUUAG A
        UU----GU-----  CA-U         G-       A

>miRNA63
  C      -C  -           C--          CU     C  -          U A           -CCUC    -     -AC -UU   UG
GG AGAGGA  UG GCCUGGCAUGG   GAUGGAAGCU  GCUU GG UAGCCAAGGA G GCUGCCUGUGG     CUGC UGCGG   G   GCG  G
CU UCUCCU  AC CGGAUCGUACC   CUACCUUCGG  CGAG CU AUCGGUUCCU C CGACGGACGCC     GGCG GCGCC   C   CGC  C
  -      CU  U           -UC          --     U  G          - -           CCU--    U     AC- UC-   CC
```

```
>miRNA64
--              C-          A-          AUA-GAUU
  GAGGUAUUGGCGCG  CUCAAUCCGA  GGCGUGGCUG        G
  CUCUAUAACUGCGC  GAGUUAGGUU  UUGUACCGAC        |
CC              -C          -C          --GGCGCG

>miRNA65
      A  A        C C  C      A   ACU- A-       GC
UGGAGG AG GAUAUUGG G GG UCAAUC GAU    G  UUUUACA  U
ACCUUU UC CUAUAACC C CC AGUUAG CUA    C  AAAAUGU  |
      C  G        A U  A      G   CAAU -C       UU

>miRNA66
             GCA           C      A   AA   A
UGACCAAUCCAUA   UGGUGCUCACU UCUUCU UCA  UUG A
GCUGGUUAGGUAU   ACUACGAGUGA AGAGGA AGU  GGC G
             AGG           U      A   C-   C
```
